# A decade of radiomics research: are images really data or just patterns in the noise?

**DOI:** 10.1007/s00330-020-07108-w

**Published:** 2020-08-07

**Authors:** Daniel Pinto dos Santos, Matthias Dietzel, Bettina Baessler

**Affiliations:** 1 Institute of Diagnostic and Interventional Radiology, University of Cologne, Medical Faculty and University Hospital Cologne, Kerpener Str. 62, 50937 Köln, Germany; 2 Institute of Radiology, University Hospital Erlangen, Maximiliansplatz 3, Erlangen, 91054 Germany; 3 Institute of Diagnostic and Interventional Radiology, University Hospital Zurich, Rämistrasse 100, Zurich, 8091 Switzerland

## Abstract

*• Although radiomics is potentially a promising approach to analyze medical image data, many pitfalls need to be considered to avoid a reproducibility crisis.*

*• There is a translation gap in radiomics research, with many studies being published but so far little to no translation into clinical practice.*

*• Going forward, more studies with higher levels of evidence are needed, ideally also focusing on prospective studies with relevant clinical impact.*

Since Lambin et al first coined the term radiomics in early 2012, almost a decade has passed [1, 2]. At that time, medical imaging and automated image analysis had already seen significant advances (and certainly have seen more innovation since then), and the concept seemed promising. In radiomics research, radiological image data are processed in order to extract large amounts of quantitative image features, which are subsequently analyzed to identify meaningful patterns and novel imaging biomarkers [3]. In most cases, radiomics is applied to oncological imaging, e.g., to support discrimination of histological tumor subtypes, predict treatment response, and consequently support more individualized therapy regimes [4]. Understandably, research interest has been unbroken since then, and numerous studies have been published discussing the application of radiomics in various settings (Fig. 1). Coming close to a decade of research in radiomics, it might be worthwhile taking a look at what results have been achieved and what has been translated into clinical use.

**Fig. 1:**
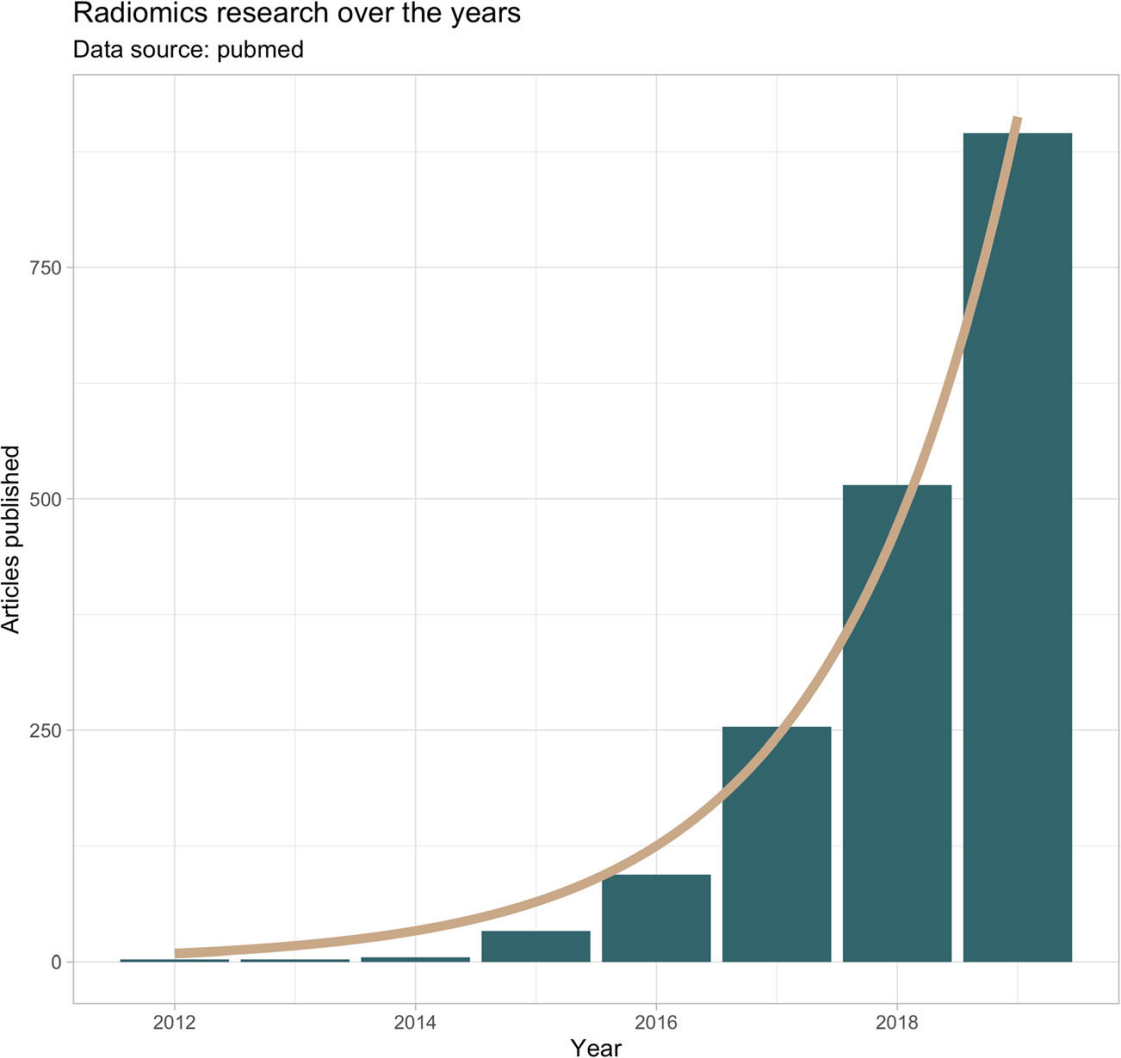
Number of articles published (until 2019–2020 was excluded at the time of writing due to incomplete data) matching the search term “radiomics” on PubMed (https://pubmed.ncbi.nlm.nih.gov/?term=radiomics)

At first sight, it seems that radiomics research could potentially have a huge impact on clinical routine. Recently, various studies addressing interesting clinical scenarios have been published. For instance, in a study published by Cui et al, a radiomics model was proposed to predict complete response to chemoradiotherapy in patients with locally advanced rectal cancer [5]. In another study, Baessler et al showed that a radiomics model could potentially differentiate between benign and malignant lymph nodes after chemoradiotherapy for metastatic germ cell tumors [6]. In both studies, it could be argued that radical oncological resection (i.e., proctectomy in the first, and retroperitoneal lymph node dissection in the latter case) could carry significant peri- and postoperative morbidity. Hence, reducing overtreatment by incorporating the results of such radiomics models in clinical decision-making could be beneficial—if not in all, then at least in selected cases. Nevertheless, it seems that despite the considerable number of publications on the subject, translation of such encouraging findings into clinical application is yet to happen. There are certainly various factors that play a role here, but a few important challenges stand out, which should be considered in future radiomics studies (Table 1).

**Table 1:** Key considerations for study design

Selected features	Should clearly be described, name selected features, ideally along with information on how those were calculated. Instances where features like first order statistics such as “max volume of ROI” or “mean value in ROI” are selected do not necessarily qualify as radiomics and could also be obtained by “traditional” measurements.
Model building	Careful consideration of all potential pitfalls limiting the model’s generalizability (i.e., inter- and intra-reader testing of the segmentations, testing for test-retest stability, homogenizing gray level intensities and pixel spacing) and statistical validity is needed.
Selected model	The potentially best model should clearly be described. E.g., for simple models give equation with features and parameters, for more complex models make model accessible to other researchers so that results can be independently validated or further studies can be built upon those.
Model validation	Selected models should be validated on an independent test set that was set aside before training the algorithm. Sampling method used should be clearly described (e.g., randomized stratified sampling).
Model performance	Clinically meaningful performance metrics should be provided, such as PPV, NPV or F metrics. Depending on the incidence of disease even with a high AUC/sensitivity/specificity the PPV might be low, thus limiting its clinical usefulness. Also, the model should be benchmarked in comparison to expert human readers or models built on known clinical characteristics (e.g., size, LIRADS, laboratory results).
Intended clinical use	It should be clearly stated which purpose the model could serve and what clinical need it serves. E.g., while a model might discriminate between two tumor stages, if no potential of stratification (due to missing alternatives or disproportionate risk of harming the patient if treatment is changed) is given, clinical usefulness may be limited.
Impact on outcome	If a proposed radiomics model has been previously described and its results are reproducible and validated, outcome studies should carefully be designed to prove the model’s utility in clinical practice aiming to benefit the patient’s outcome. In order to move the field forward, there is an urgent need for prospective trials to demonstrate the impact of radiomics on patient outcome.

Although radiomics research holds the potential to positively impact patient management and outcome, scientific rigor is needed in order to prove the benefits beyond reasonable doubt [7]

First of all, a significant proportion of the published studies on applications of radiomics are of insufficient quality. This is of course a bold statement and should not be said lightly. Recently, Park et al carried out a detailed analysis of multiple studies and assessed their methodological quality using the radiomics quality score (RQS), as well as how results were reported according to the Transparent Reporting of a multivariable prediction model for Individual Prognosis Or Diagnosis (TRIPOD) checklist [8]. The results were—to put it mildly—sobering. With a mean RQS score of only 26.1% and a mean adherence rate to the TRIPOD checklist of only 56.8%, there is obviously a lot room for improvement. These findings should, however, not be intended to devalue existing research, but rather be taken as an eye-opener encouraging us to strive for the highest possible scientific rigor—from the design of the studies all the way through the review and publication process. An open, self-reflecting discussion may be needed to analyze, why and how such findings come to be. Among the first steps, one possible approach could be to require authors and reviewers to follow checklists such as the aforementioned or the recently proposed Checklist for Artificial Intelligence in Medical Imaging [9].

Secondly, given that most approaches to radiomics rely on the analysis of distribution of gray values in a specified region or volume of interest (ROI/VOI), the inherent problem of medical imaging needs to be carefully considered. For example, while one specific scanner might lead to reproducible gray value distributions in a single patient at a single time point when the analysis is carried out by a single reader, this is not necessarily the case when another patient with the exact same pathology is scanned on a different machine, the same patient is scanned at different time points or even when different readers assess the images and place the ROI/VOI. In the worst cases, we as radiologists might be able to see the patterns beyond the noise, but an algorithm that performs complex calculations might easily be derailed by just the tiniest amount of noise [10, 11]. To further add to this complexity, in most cases, complex statistical approaches and machine learning are used to build prediction models based on radiomics features which come with their own challenges [12, 13]. To tackle these issues and avoid getting lost in a reproducibility crisis, careful methodological and statistical consideration of potential pitfalls is crucial [7, 14].

Lastly, in order to close the “translational gap” of radiomics, it will be crucial to obtain higher evidence levels and move beyond exploratory retrospective studies. Carefully designed prospective, multicenter, randomized controlled trials and data sharing will be needed in the future to prove the clinical usefulness of radiomics and subsequently improved patient outcomes in a setting as close to clinical routine as possible [15, 16].

Of course, neither was Rome built in one day, nor did cardiac CT find its way to clinical routine just shortly after the first developments in 1976 [17]. Nevertheless, in order to move the field of radiomics forward, future research should focus on the challenges mentioned above (Table 1). It might not be an easy task, but the effort could prove worthwhile—or as a prominent political figure might have said, had he done research in radiomics: “We should choose to bring radiomics to clinical routine in this decade, not because it is easy, but because it is hard; because the goal should be to serve our patients and improve outcomes”.
